# The Neuroprotective Potentiality of Flavonoids on Alzheimer’s Disease

**DOI:** 10.3390/ijms232314835

**Published:** 2022-11-27

**Authors:** Antonella Calderaro, Giuseppe Tancredi Patanè, Ester Tellone, Davide Barreca, Silvana Ficarra, Francesco Misiti, Giuseppina Laganà

**Affiliations:** 1 Department of Chemical, Biological, Pharmaceutical and Environmental Sciences, University of Messina, Viale Ferdinando Stagno d’Alcontres 31, 98166 Messina, Italy; 2 Department of Human Sciences, Society and Health, University of Cassino and Southern Lazio, V. S. Angelo, Loc. Folcara, 3043 Cassino, Italy

**Keywords:** flavonoids, neuroprotection, quercetin, myricetin, epicatechin-3-gallate, naringenin, cyanidin 3-o-glucoside, apigenin, genistein, gossypetin

## Abstract

Alzheimer’s disease (AD), due to its spread, has become a global health priority, and is characterized by senile dementia and progressive disability. The main cause of AD and other neurodegenerations (Huntington, Parkinson, Amyotrophic Lateral Sclerosis) are aggregated protein accumulation and oxidative damage. Recent research on secondary metabolites of plants such as polyphenols demonstrated that they may slow the progression of AD. The flavonoids’ mechanism of action in AD involved the inhibition of acetylcholinesterase, butyrylcholinesterase, Tau protein aggregation, β-secretase, oxidative stress, inflammation, and apoptosis through modulation of signaling pathways which are implicated in cognitive and neuroprotective functions, such as ERK, PI3-kinase/Akt, NFKB, MAPKs, and endogenous antioxidant enzymatic systems. This review focuses on flavonoids and their role in AD, in terms of therapeutic potentiality for human health, antioxidant potential, and specific AD molecular targets.

## 1. Introduction

Alzheimer’s disease (AD) is the most common cause of senile dementia associated with progressive disability. The inherited disease, in an autosomal dominant way, generally leads to a lethal outcome after about 5–10 years from the onset of the first symptoms [1]. Its pathology is complex, characterized by a decline in memory that, in its most common form, arises after 60 years of age. More rarely, the symptomatology can begin between 40 and 50 years, and in this case, the disease has a very rapid progression [2]. Generally, the early-onset form, called “familial”, is related to specific mutations in the genes encoding presenilin1 (PS1) and 2 (PS2) and amyloid precursor protein (APP), while sporadic late-onset disease is associated with mutations in the gene encoding apolipoprotein E (ApoE), and includes several environmental risk factors. [3]. The exact etiology of AD is not yet known, but several mechanisms have been described, including cholinesterase deficiency and generation of oxidative stress [4,5]. The histological features of AD are the extracellular deposits of the amyloid beta peptide (Aβ), in the form of neuritic plaques. The intraneuronal neurofibrillary tangles (NFTs) constitute aggregates of hyperphosphorylated Tau protein [6]. The symptomatology is characterized by an initial difficulty with language, concentration, and orientation that evolves into motor difficulties and personality changes, leading to a serious impact on public health and a strong burden on the field of health [7]. To date, approximately 50 million cases of AD have been estimated in the world (this number will more than double by 2050) and it is predicted to double every 5 years. Several drugs have been selected to combat the disease, but unfortunately, due to the different natures of the pathological targets related to the progression of AD, none of these modify the disease; they confer only milder management and transient symptoms. Further studies are needed to better characterize the risk factors that predispose one to the progression of AD, and to identify drugs to counteract the evolution of the disease and/or defend against its development. In this context, diet and natural products are show great promise in helping to reduce the development of neurodegenerative diseases [8,9,10,11,12,13,14,15,16]. In fact, unlike synthetic products, which possess serious side effects, the use of natural compounds can be a good alternative therapy. The purpose of this review is to describe the state of the art of current knowledge on AD and on the biochemical targets that trigger its pathological progression, as well as to highlight the potential protective association of flavonoids for the purpose of reversing the age-related decline caused by AD.

## 2. Oxidative Stress and Alzheimer

**Figure 1:**
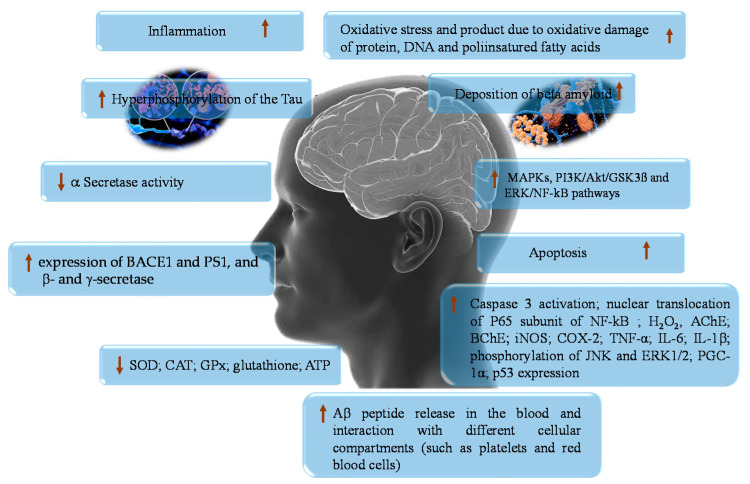
Schematic representation of the main features of Alzheimer’s disease. (↑ increase; ↓ decrease).

Oxidative stress is defined as an imbalance between oxidants and antioxidants that causes a rise in oxidant levels [17]. According to the amyloid cascade hypothesis, accumulation of non-soluble amyloid β peptides in the Central Nervous System (CNS) is the primary cause that initiates a pathogenic cascade, leading to the complex multilayered pathology and clinical manifestation of the disease. It is, therefore, not surprising that the search for mechanisms underlying cognitive changes observed in AD has focused on the brain and Aβ-inducing oxidative stress. However, since Aβ depositions can be found in normal, non-demented elderly people and in many other pathological conditions, the amyloid cascade hypothesis was modified to claim that intraneuronal accumulation of soluble Aβ oligomers, rather than monomer or insoluble Aβ fibrils, is the first step of a fatal cascade in AD (Figure 1). Oxidative stress was initially proposed to be a major factor in AD in 1986 [18]. Overwhelming evidence exists that the cells in the Alzheimer’s brain undergo abnormally high levels of oxidative stress, and that those amyloid plaques are a focus of cellular and molecular oxidation [19]. Aβ peptides trigger oxidative stress in the brain [20,21]. In addition to mediating Aβ-induced cytotoxicity, numerous studies have suggested that oxidative stress promotes the production of Aβ. It has been demonstrated that defects in the antioxidant defense system caused elevated oxidative stress [22,23]. Previous studies have shown that oxidative stress decreases the activity of alpha-secretase while promoting the activation of a cascade of redox-sensitive cell signal pathways, including JNK, which promotes the expression of BACE1 and PS1, and eventually β- and γ-secretase, enzymes critical for the generation of Aβ from APP [24,25]. Notably, the oxidative damage appears to become pronounced following the interaction of the sulfur-free radical with methionine 35 in the Aβ peptide [26]. The brain in AD appears to sustain more oxidative damage than normal, with low levels of antioxidants [27,28].

Therefore, while the brain membrane phospholipids are composed of polyunsaturated fatty acids, this organ is particularly vulnerable to free radical attacks. Plasma levels of thiobarbituric acid are high in the early stages of AD [29]; lipid hydroperoxides are the unstable products of lipid peroxidation, and they undergo non-enzymatic decomposition to generate aldehydes such as malondialdehyde (MDA) and 4-hydroxynonenal (4-HNE). High blood hydroperoxide levels are associated with mild cognitive impairment (MCI) and AD [30].

Proteins are major targets of reactive oxygen species (ROS). Protein oxidative modifications can induce unfolding or conformational changes that can lead to the loss of specific protein function [31] and the formation of cross-linked protein aggregates, which are resistant to removal by proteinases. Increased reactive oxygen species production and oxidative modification of brain proteins are significant in AD pathogenesis [32]. Carbonyl formation of 3-nitrotyrosine (3-NT) is an important marker of protein oxidation. Protein carbonyls and 3-NT levels were increased in the frontal cortex of individuals with MCI, mild AD, and AD [33].

Nucleic acid damage also occurs early in AD. Significantly elevated levels of 8OHG and 4,6-diamino-5-formamidopyrimidine have been reported in post-mortem MCI brains relative to the age-matched controls [34]. In addition to oxidative damage, reduced antioxidant defenses have been reported in MCI and early AD [35,36,37]. Plasma glutathione levels and antioxidant enzymes, such as glutathione peroxidase, catalase, and superoxide dismutase (SOD), are significantly decreased in early AD [29,38].

Aside from its presence in CNS, Aβ can be detected in platelets [39] and blood [40], where it interacts with red blood cells (RBC). Previous studies [41,42,43] suggest that Aβ-induced oxidative stress alters RBC metabolism.

## 3. Flavonoids

Recent studies have tested the power of natural compounds derived from plants against AD. Among these, flavonoids are ubiquitous compounds of plants, produced by plants for growth and defense against all kinds of stress, including cold tolerance. More than 6000 different flavonoids have been identified, the primary sources of which are apples, red fruits, onions, citrus fruits, nuts, and beverages such as tea, coffee, beer, and red wine. These compounds, derived from phenol, are particularly interesting for their ability to cross the blood–brain barrier and for their multi-target activity. Several studies have described flavonoids to exhibit relevant biologic activities involving the neuronal antioxidants, as well as anti-amyloidogenic properties, acting as metal chelators, showing anti-inflammatory properties, and ameliorating cognition and neuroprotection [44,45,46,47,48,49,50,51,52]. All of these capabilities are critical to counteract neurodegeneration, as they help to safeguard the number and efficiency of neurons as well as the integrity of their synaptic connections. Epidemiological studies have highlighted a direct relationship between diets rich in flavonoids, reduced risk of dementia, and potential for improved symptomatology in patients with AD [53,54]. Several subclasses of flavonoids have been identified; all are present in the human diet, as they are very abundant in vegetables, fruits, and some beverages [55]. In general, we can find flavonoids in all parts of plants, since they are produced by the plant itself for its growth and defense against all kinds of stress, including cold tolerance [56,57].

### 3.1. Chemical Structure and Flavonoids Classification

**Figure 2:**
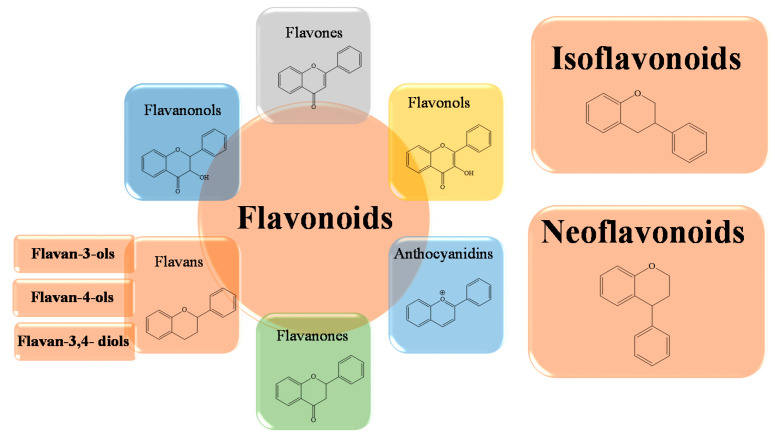
Schematic representation of the basic structures of flavonoid subclasses.

From a chemical point of view, flavonoids consist of two benzene rings, called A and B, linked via a third pyranosic ring C. Flavonoids can be divided into a variety of subclasses that differ in terms of the structural characteristics of the B ring and the degree of hydroxylation and glycosylation of the third ring. Typically, ring B binds in position 2 on ring C, but can also bind in position 3 or 4. We can, therefore, distinguish the isoflavones in which the B ring binds in position 3 of the C ring and the neoflavonoids in which the B ring binds in position 4 of the C ring (Figure 2). The group of flavonoids in which the B ring is linked in position 2 of the C ring can be further divided into six subgroups, according to the structural peculiarities of the C ring: flavones, flavonols, flavanones, flavanonols, flavanols, catechins, and anthocyanins [58]. Finally, the flavonoids in which the C ring is open are called chalcones.

In general, since all flavonoids contain the same core scaffold, the functional differences between the various groups and subgroups are mainly due to the different substituent groups. These are weak polybasic acids of a polyphenolic nature, characterized by varying degrees of hydroxylation, methoxylation, glycosylation or glucuronidation, and this contributes to the great variety of biological properties of this large group of polyphenols [59,60]. In fact, a different side chain can significantly influence the activity of flavonoids on the same molecular target, and the total number of hydroxyl groups is important for the enhancement of antioxidant activity, free radical scavenging, and metal ion chelation [61,62,63,64].

### 3.2. Biological Activities of Flavonoids

Flavonoids are a wide group of secondary metabolites characterized by many interesting biological potentials, both in vitro and in vivo (such as anticancer, antioxidant, antiaging, anti-inflammatory, antimicrobial, and immunomodulatory activities, along with modulation of the activity of key metabolic enzymes, cytoprotective and cardioprotective potentials, and inhibition of cellular proliferation, for example) and, in the last decades, they have emerged as a promising agents for neuroprotection [58,65,66,67,68]. In a recent epidemiologic study, Shishtar et al. [69] analyzed long-term dietary flavonoid intake and risk of Alzheimer’s disease and related dementias in the Framingham Offspring Cohort, with a total of 5209 participants aged 28–62 years in the original cohort. The intake effects of six classes of dietary flavonoid (flavonols, flavones, flavanones, flavan-3-ols, anthocyanins, and flavonoid polymers) and the risk of Alzheimer’s disease and related dementias (ADRD) and Alzheimer’s disease (AD) alone were analyzed based on the data from the Framingham Heart Study Offspring Cohort. Participants were ADRD-free with a valid FFQ at baseline. Flavonoid intakes were updated at each exam in order to represent the cumulative average intake across the five exams, and were expressed as percentile categories of intake to handle their nonlinear relation with ADRD and AD. After multivariate and dietary adjustments, individuals with the highest intakes of flavonols, anthocyanins, and flavonoid polymers had a lower risk of ADRD relative to individuals with the lowest intakes. A similar trend was found also in AD for flavonols and anthocyanins, but not for flavonoid polymers, showing a direct correlation between higher long-term dietary intake of flavonoids and the risks of ADRD and AD onset in American adults.

Flavonoids are widely distributed in the plant kingdom, and are characterized by chemical structures with the presence of several substituents that allow them to assume particular activities and exert beneficial effects for the wellness of organisms, as well as for their potential for therapeutic utilization. This does not indicate that every type of flavonoid is able to show biological potential, but only that those with particular characteristics can be employed for specific roles. For instance, one of the well-known and best-studied activities of flavonoids is its antioxidant activity, which is linked to the number of hydroxyl groups on the B ring. Generally, a greater number of free hydroxyl groups corresponds to a greater scavenging effect, but their location in the skeleton of flavonoids is a crucial structural element. These hydroxyl groups, through the donation of hydrogen atoms and electrons to radical species, favor the repair of the damage caused by ROS and reactive nitrogen species (RNS), reducing their degree of reactivity [70,71]. This mechanism leads to the generation of a relatively more stable flavonoid radical, which significantly reduces the oxidative stress triggered by the interrupting free radicals. At the level of the cell membrane, there is a chain reaction of propagation of peroxylic radicals between the molecules of polyunsaturated fatty acids and other intermediates [72,73]. The scavenger action of flavonoids on ROS and RNS is important because the ROS/RNS balance is directly connected with the redox state of the cell, which is also influenced by the presence of metal ions. In addition to the direct action on free radicals, experimental data demonstrate specific chelating properties of flavonoids against transition metals, mainly iron and copper ions [74,75,76]. Ferrous and/or copper ions represent a danger because they tend to react through the Fenton reaction, with hydrogen peroxide generating hydroxyl radicals, a very reactive species which rapidly oxidizes surrounding molecules, triggering the oxidative stress cascade. Chelating activity further enhances the ability of flavonoids to protect against oxidative stress, since the chelate metal ions may not participate in the generation of ROS through the Fenton reaction and because the chelates have a more powerful scavenger action against ROS than free flavonoids [77,78,79]. The chelating activity of flavonoids is considered a key mechanism for the biological activity of flavonoids, because metallocomplexes affect several biochemical properties, such as lipophilicity, membrane transport, and interaction with biomolecules [80,81,82]. In a second antioxidant mechanism, flavonoids do not act directly on ROS, but “indirectly” interact with some proteins involved in the gene expression regulation pathway. They upregulate the endogenous antioxidant capacity of the cell, and inhibit others involved in redox balance and inflammatory processes, such as cyclooxygenase, lipoxygenase, xanthine oxidase, NADH oxidase, and myeloperoxidase [83,84,85,86,87,88,89]. Therefore, the mechanisms we have described suggest not only a direct involvement of the flavonoid molecule, but also of metabolites that result from its oxidation and the formation of flavonoid–metal complexes [90].

### 3.3. Flavonoids in Neurodegeneration

The ability of flavonoids to cross the blood–brain barrier suggests that these compounds can feasibly have a direct effect on the brain. Numerous studies have documented the bioactivity of flavonoids against neurodegenerative disorders such as AD, Parkinson’s, Huntington’s, and other neurological disorders [51,58,64,65,66,67,68,69,91,92,93]. Regarding AD treatment, there is still no significantly efficient drug that can reduce the progression or improve the outcome of the disease [94,95]. The search for natural substances for the treatment of AD is considered key to brain health, because these compounds are often easily isolated and possess well-documented biomechanisms and safety profiles [96,97]. In addition, their abundance in vegetables and fruits has made them a major part of the human diet.

### 3.4. Potential Role of Flavonoids in AD Therapy

**Figure 3:**
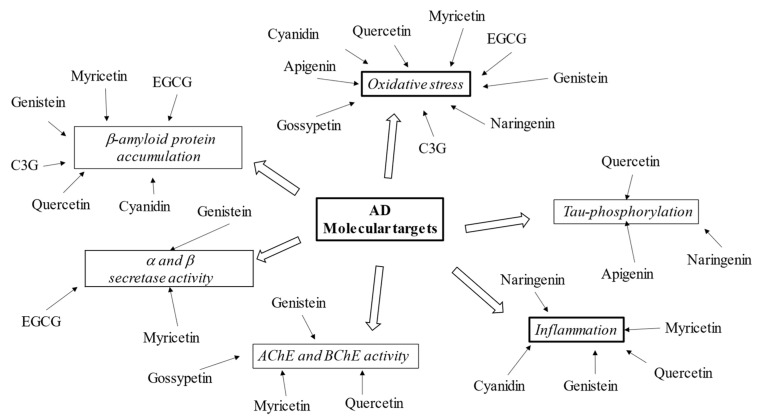
Schematic effects of described flavonoids on molecular targets of AD.

Flavonoids, including epicatechin-3-gallate, gossypetin, naringerin, quercetin, and myricetin are reported to block β-amyloid and Tau aggregation, scavenge free radicals, and sequester metal ions at clinically low concentrations [98,99]. In order to better understand flavonoids’ role in AD treatment, the pharmacological effects of some compounds from the six subclasses are described (see Figure 3).

Quercetin is a polyhydroxyflavonoid that belongs to the subclass of flavonols. Its chemical name is 3,3,4,5,7-pentahydroxyflavone, and the molecule contains five -OH groups, in positions 3, 3′, 5, 7 and 4′, which are crucial for potential biochemical–pharmacological activities. Quercetin is a natural antioxidant, widely used in healthcare for its beneficial role. Quercetin is found in flowers and fruits of edible plants; onions, apples, cherries, berries, asparagus, and red leaf lettuce have the highest levels, while tomatoes, peas, and broccoli have lower levels [92,93]. Experimental studies have shown the existence of an inverse correlation between dietary quercetin intake and risk of senile dementia. Specifically, experimental studies have identified various quercetin targets of neuronal protection that contribute to reducing the main neuronal lesions present in the brains of AD patients. Among them are hyperphosphorylation of the Tau protein, the deposition of beta amyloid, oxidative stress, inflammation, and apoptotic processes [100,101].

More specifically, Tau phosphorylation is under the control of several distinct kinases, such as Erk, Akt, p38, AMP activated protein kinase (AMPK), glycogen synthase kinase 3 beta (GSK3β), cyclin-dependent kinase 5 (cdk5), and protein phosphatase 2A (PP2A) [102,103]. Through molecular dynamic simulation studies carried out according to the molecular docking results, Zu et al. identified MAPK as core target of quercetin. Jiang et al. demonstrated the anti-apoptotic role of quercetin via MAPKs and PI3K/Akt/GSK3ß signaling pathways by preincubating HT22 cells with 5 μmol/L of the drug for 12 h [103,104]. MAPK is a heterotrimeric Ser/Thr protein kinase; its activation contributes to the hyperphosphorylation of Tau in neurons. It also checks Aβ metabolism, and is involved in cell proliferation, apoptosis, and inflammatory responses [105]. Thus, MAPK pathway inhibition can significantly improve synaptic plasticity, memory, and cognitive functions, and can be considered a valid target against AD progression [106]. GSK3β, a Ser/Thr kinase that connects numerous signaling pathways in the cell, including inflammatory responses, is another strategic target for neuroprotection. GSK3β connects numerous signaling pathways in the cell. It is a downstream enzyme of the PI3K/Akt signaling pathway, and is considered the main factor responsible for the phosphorylation of the Tau protein in AD [107]. Quercetin acts on GSK3β, decreasing the kinase activity, and therefore, quercetin indirectly has an anti-hyperphosphorylation of Tau protein action that helps to strengthen its neuroprotective effects [104]. Bao et al. have demonstrated that pretreatment for 2 h of rat pheochomocytoma PC-12 cell line with 500 µM quercetin attenuates H_2_O_2_-induced p53 expression, and also significantly reduces apoptosis and caspase 3 activation [108]. p53 regulates the action of nitric oxide synthase (NOS) and represses the transcription of peroxisome proliferator-activated receptor-γ coactivator (PGC-1α), one of the most powerful stimulators of mitochondrial biogenesis and respiration [109,110]. In AD, the decrease in the expression of PGC-1α is one of the causes related to the progression of the disease, as it is linked to the increase in the generation of the peptide Aβ [109,110]. In addition, the quercetin action on p53 affects the oxidative stress decrease, because p53 is a significant activator of the ROS-mediated apoptotic pathway [111,112]. A total of 50 μM quercetin, after 4 h of HepG2 cells incubation, has been shown to restore cellular redox homeostasis through its significant scavenger activity against ROS. Feeding the C57BL/6J mice with a 1% quercetin diet for 20 weeks increased the level of glutathione (GSH) and the expression of certain antioxidant enzymes, including SOD, catalase (CAT), and glutathione peroxidase (GPx), in hippocampal neurons [113,114,115,116].

**Figure 4:**
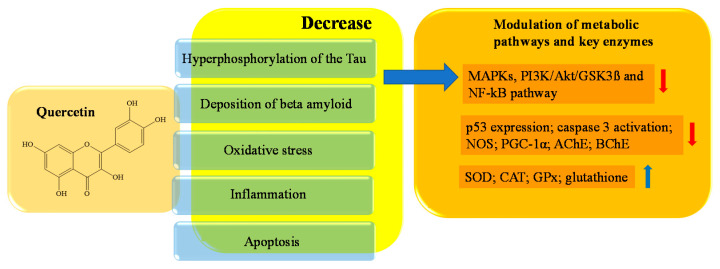
Schematic effects of quercetin on molecular targets of AD.

Hung et al. demonstrated that quercetin (10mM) pretreatment of human umbilical vein endothelial cells (HUVECs) suppressed the nuclear factor- kB (NF-kB) signal, suggesting that the drug is a powerful antiatherosclerotic [117]. In addition, quercetin is a strong inhibitor of two important key enzymes involved in the pathology of AD, namely acetylcholinesterase (AchE) and butyrylcholinesterase (BchE) [118,119]. AChE and BChE contribute to hydrolytic degradation of acetylcholine (ACh), an important neurotransmitter that coordinates the excitability and activation of groups of neurons in the brain, and also influences its transmission and synaptic plasticity [120]. In the brain, a decrease in ACh corresponds to a slowdown in communication between neurons. Quercetin, through its OH groups of the phenyl ring, forms hydrogen bonds with specific amino acids in the active site of the AChE [121]. Inhibition of AChE and BChE facilitates communication between nerves and increases the activity of cholinergic pathways in the brain, relieving symptoms of memory loss [122]. In the brain, BChE also appears to play a role in the transformation of dangerous amyloid plaques to the pathogenic structures present in dementia and AD [123,124]. In Figure 4, a schematic representation of the main activities of quercetin is depicted.

**Figure 5:**
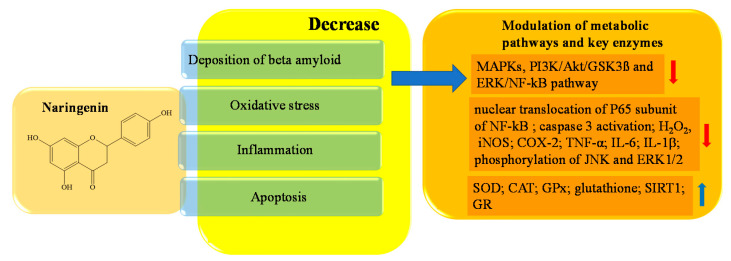
Schematic effects of naringenin on molecular targets of AD.

Naringenin is the aglycon of naringin, and belongs to the subclass of flavanones. It is abundant in citrus fruits (especially grapefruits, to which it gives the characteristic bitter taste), as well as vegetables, and especially in grapes, tomatoes, and cherries. Naringenin can be found in two forms. One is characterized by a bond with a sugar on C7, and one derives from the action of specific enzymes which are able to cleave this glycosidic bond by releasing the aglycone [125,126]. Both forms have antioxidant activity, but naringenin has a more powerful scavenging action than its precursor naringin, because the presence of sugar in the latter causes a steric hindrance that impairs activity [127]. Naringenin (400 mM) improved learning and spatial memory in PC12 cells of rats with AD through the regulation of the PI3K/Akt/GSK-3K pathway and by reducing the hyper-phosphorylation of TAU. The intracellular mechanism that allows this neuroprotective action is related to the inhibition of caspase 3 activity, the activation of PI3K/Akt, and the modulation of the signaling pathway GSK3β, which plays a crucial role in neuronal survival [128,129,130]. The inhibition of caspase 3 also affects programmed cell death; in fact, the block of neuroapoptosis is also caused by the decreased levels of malondialdehyde and hippocampal nitrite found in socially defeated rat pups treated with naringenin (50–100 mM) [131]. Naringenin significantly regulates the (NF-kB) signaling pathway, which is implicated in inflammatory processes, and decreases tumor necrosis factor-α (TNF-α) as well as interleukin (IL)-6 and IL-1β [131]. The reduced expression of NF-kB is induced by naringenin through a significant decrease in phosphorylation and nuclear translocation of P65, a subunit of NF-kB, as well as by an increase in sirtuin 1 (SIRT1) levels in the hippocampus [132,133,134,135]. The inflammatory pathway is also repressed by the interaction of naringenin with other molecular targets, including inducible nitric oxide synthase (iNOS) and cyclooxygenase 2 (COX-2) e MAPK. iNOS and COX-2 are both inhibited in a concentration-dependent manner, while Zhang et al. demonstrated that naringenin (100 mM) inhibited MAPK signaling pathway activation by suppressing the phosphorylation of JNK and ERK1/2 in BV-2 cells [136]. Numerous studies have reported the antioxidant properties of polyphenols; it has been shown that GSH activity, one of the most efficient endogenous antioxidants, is improved by naringenin. Additionally, an increase in SOD, CAT, GPx, and glutathione reductase (GR) activity, in addition to a decrease in hydrogen peroxide (H_2_O_2_) and protein carbonyls levels, have been demonstrated [137,138,139,140]. In Figure 5, a schematic representation of the main activities of naringenin is depicted.

**Figure 6:**
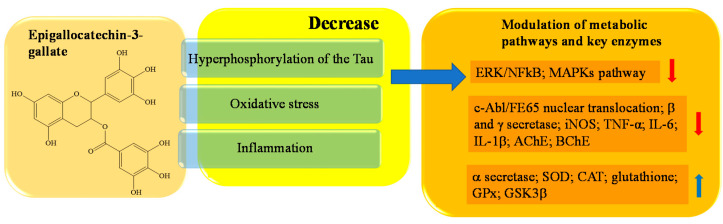
Schematic effects of epigallocatechin-3-gallate on molecular targets of AD.

Epigallocatechin-3-gallate (EGCG), an ester of epigallocatechin and gallic acid, is the main bioactive polyphenol found in solid green tea extract [141,142]. EGCG has been reported to bypass the blood–brain barrier (BBB) and exert potent neuroprotective properties against AD, in a wide range of cell models [143]. Several works have shown that EGCG (5-15 mg/kg) reduces the accumulation of β amyloid able to interfere with the formation of β-sheets, the process involved in amyloid formation cascade [144,145]. EGCG promotes the non-amyloidogenic process by promoting α secretase cleavage and inhibiting β and γ secretase, by way of suppression of the ERK/NFkB pathway [146,147]. Sonawane et al. demonstrated the EGCG’s potential for dissolving pre-formed Tau filaments and oligomers in a time- and concentration-dependent manner; the IC50 for Tau aggregation by EGCG was found to be 64.2 μM [148]. Ehrnhoefer et al. have demonstrated that EGCG can convert mature Aβ fibrils into smaller forms free of toxicity, redirecting polypeptide aggregation into off-pathway protein assemblies [145]. In APP-C99-overexpressed cultured MC65 cells, EGCG (5–20 μM) is also able to suppress Aβ-induced neurotoxicity through GSK3β activation and inhibition of c-Abl/FE65 nuclear translocation [149]. In addition, it attenuates oxidative stress and mitochondrial impairment, and restores intracellular antioxidant levels in different neuronal cell lines and AD models [150]. More specifically, in EOC 13.31 microglial cell lines, EGCG (5–20 μM) suppresses the expression of TNFα, iNOS, IL-1β, and IL-6, and it inhibits the activation of the NF-Kβ and MAPK signal, but increases the synthesis of GSH. Chi et al. demonstrated significant protection of EGCG (22.5–90 μM) against oxidative damage caused by H_2_O_2_ to chicken lymphocytes. After preincubation with EGCG, the compound restored H_2_O_2′_s harmful effects, suppressing the increase of ROS and restoring the antioxidant system by mRNA expression of SOD, CAT, and GPx [151]. EGCG enhances cholinergic neurotransmission through inhibition of AChE and BChE. ACh content was significantly elevated in a dose-dependent manner, which ultimately led to an improvement in the learning and memory function of AD rats [152]. In Figure 6 a schematic representation of the main activity of epigallocatechin-3-gallate is depicted.

**Figure 7:**
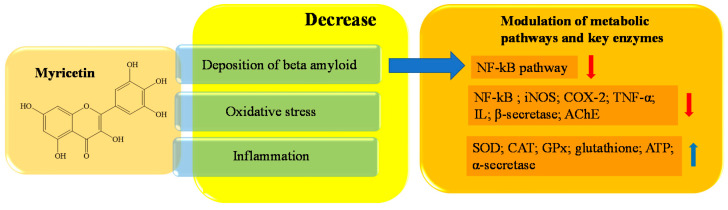
Schematic effects of myricetin on molecular targets of AD.

Myricetin is a natural flavanol widely distributed in several vegetables and fruits, mainly including blackcurrant teas, red wines, and medical herbs [153,154,155,156]. Myricetin, mainly in the form of glycoside (O-glycosides), is also known as hydroxy quercetin because of its quercetin-like structure [157,158], from which it differs by one extra hydroxyl at the 5′-OH of the B ring. The compound has a wide range of beneficial effects on human health, including antihypertensive, antiallergic, analgesic, anti-inflammatory, immunomodulatory, antiplatelets, and aggregation activities [158,159,160]. Several studies have indicated the neuroprotective properties of myricetin, which are expressed through different molecular targets. Ramezani et al. have shown that intraperitoneal injection of myricetin, at a dose of 5 or 10 mg/kg over 21 days, improves learning and memory in rat models with AD [161]. Myricetin exhibits antiamyloidogenic activities; it reduces the formation of ordered Aβ aggregation through the formation of H-bonds between its hydroxyl group and the carbonyl group on the surface of the β sheet [162,163,164]. In this way, myricetin weakens the interstrand hydrogen bonds, inhibits the extension of fibrils of Aβ, and prevents Aβ from undergoing toxic changes [164,165,166]. Antiamyloidogenic activity is also supported by the compound’s ability to interact with α and β-secretase. In more detail, in cultured rat primary cortical neurons, myricetin (10 μM) has been shown to increase the α-secretase (ADAM10) level and enzyme activity, while it inhibits the activity of β-secretase (BACE-1) with an IC50 of 2.8 µM [167,168]. Chakraborty et al. have shown that an H-bond is created between the hydroxyl group in position C7 of the A ring of myricetin and the dyad of Asp 32 and 228 of BACE-1; in this way, the enzymatic activity is strongly reduced [168]. Moreover, myricetin shows significant antioxidant and free radical scavenging effects [156,157,159,169]. It has been reported that in murine models, myricetin (40 and 80 µM) can inhibit oxidative stress generation of ROS and myeloperoxidase, and depletion of glutathione and ATP. However, it restores the levels and activity of the main antioxidant enzymes, such as SOD, CAT, and GSHpx in animal models [159,170,171]. Treatment with 80 μM myricetin for 3 h increased cell viability to 81% ± 4.2% of isolated cardiomyocytes intoxicated with 20 μg/mL aluminum phosphide (AlP) [171]. The antioxidant power of myricetin is attributable to the pyrogallol group. The molecule tends to react with free radicals to form radical semiquinones; this ability helps to interrupt the chain of reactions triggered by ROS [172,173,174]. In addition, the compound has chelating properties on metal ions such as Cu^2+^ and Fe^2+^; this, on the one hand, strongly enhances its antioxidant activity, because the Fenton reaction is inhibited and, consequently, the ROS generation is reduced. On the other hand, myricetin acts directly on Aβ complexes, reducing their toxicity through the reduction in metal ions that can interact with them [175,176,177]. Myricetin inhibits lipopolysaccharide (LPS)-induced neuroinflammation, as it reduces the levels of proinflammatory mediators, including IL, NF-kB, TNFα, iNOS and COX2, in the microglia BV2 cell line. [178]. Finally, other myricetin-like polyphenols, including gossypetin, also has inhibitory abilities against AChE [179,180]. In Figure 7, a schematic representation of the main activity of myricetin is depicted.

Gossypetin (3,5,7,8,3′,4′-hexahydroxy flavone) is a flavonol isolated from the flowers and the calyx of Hibiscus sabdariffa. Gossypetin has been shown to exert antioxidant, antimutagenic, antimicrobial, and anti-atherosclerotic activities [181,182,183,184]. Chen et al. [183], in murine macrophage cell line J774A.1, demonstrated that gossypetin (1–1000 μM) has inhibitory effects on both lipid peroxidation and lipoprotein oxidation, attenuating the formation of foam cells and lipid accumulation through PPAR pathways. The drug improves cholesterol removal from macrophages and delays atherosclerosis [183]. In addition, Lin et al. [184] demonstrated that gossypetin (0.1–0.5 μM) has inhibitory effects on abnormal vascular smooth muscle cell proliferation and migration, which could lead to the containment of atherosclerosis and other cardiovascular illnesses [184]. Inhibition of AChE and BChE activity, key enzymes in brain-related disorders, further emphasizes the therapeutic benefit of gossypetin for the treatment of AD [180,185].

Genistein (4′,5′,7-trihydroxyisoflavone) is an isoflavone distributed in several vegetables such as legumes, green peas, and peanuts, and is predominantly extracted from the Glycine max soybean [186,187]. Several researches point out genistein potential therapeutic role to delay the onset of Alzheimer’s dementia through the improve of cognitive function and synapse development [188]. In this context, Safahani et al. demonstrated that genistein supplementation modulates dopaminergic and cholinergic function, helping with memory recovery and neuroprotection in rats [189]. Genistein (10 mg/kg) protects against AD progression by reducing the production and deposition of Aβ aggregates, as well as the hyperphosphorylation of the Tau protein, in rat models [188,190,191]. Genistein protects against AD progression by reducing the generation and aggregation of Aβ. Seong et al. showed that the inhibitory activity (%) of genistein against the extent of Aβ25–35 self-aggregation, after 24 h of incubation, decreased by 34.90% when co-treated with 100 µM genistein [192]. In rat hippocampal neurons, the drug (0.375 µg/mL) downregulated presenilin levels, increased α secretase while decreasing β secretase and BACE1 activity, and, last but not least, modulated the PPARγ receptor to upregulate ApoE production [193,194,195,196]. In vitro genistein (0.391 mM) has been effectively proved to reduce oxidative stress, due to its high antioxidant power and potent ROS scavenger ability [197].

The antioxidant effects of genistein are associated with AMPK activation and the drug’s binding with estrogen receptor α (ERα), both of which promote the expression of antioxidant enzymes such as SOD, CAT, and GPx [198,199,200]. Moreover, in RAW 264.7 cell model, genistein (20 mM) prevents neuro-inflammation by regulating gene transcription of cytokines, such as TNFα, IL-1β, IL-6, and IL-12 [201,202]. Finally, Fang et al., 2014, demonstrated the inhibitory activity of AChE by two genistein derivatives, highlighting the therapeutic potentiality of this isoflavone [203].

Apigenin (4′,5,7-trihydroxyflavone) formally belongs to the flavone subclass, and is widely distributed in the plant kingdom, present principally in chamomile flowers. and in lower concentrations in vegetables, citrus fruits, herbs, and plant-based beverages (tea, beer, and wine) [204]. In a rat model of AD, apigenin (50 mg/kg) significantly reduced the hyperphosphorylation of tau levels in the hippocampus, decreasing the expression of GSK-3β, suppressing BACE1 expression, and supporting an antiamioloidogenic activity [205]. The drug (25 µM in human THP-1 monotypic cells) inhibits the production of IL-6 and IL-1β by modulating the MAPK/ERK and PI3-K/Akt signal transduction pathways associated with neuronal survival blocking [206,207,208]. Apigenin decreases ROS levels and significantly increases GSH levels, improving the cellular antioxidant defense system [209,210].

Cyanidin 3-O-glucoside belongs to the anthocyanins class; it is present in plants such as berries and soybean fruits, where it is responsible for the red, purple, and blue pigments. Cyanidin has been reported to act as neuroprotector in several disorders, such as AD, Parkinson’s disease, and multiple sclerosis [211,212,213,214,215]. The anti-AD effect is evidenced in the inhibition of Aβ amyloid accumulation. Cyanidin could directly interact with Aβ peptide through hydrogen bonds responsible for Aβ aggregation and fibrils formation [216]. In addition, Thummayot et al. (2014, 2016) demonstrated that, in human neuroblastoma (SK-N-SH) cells, cyanidin (20 μM) decreases Aβ-induced apoptosis of SK-N-SH cells by decreasing the expression levels of several proteins. This facilitates the release of the pro-apoptotic factors required for caspase cascade activation [217,218]. The neuroprotective effects of cyanidin may also be mediated via inhibition of oxidative stress and pro-inflammatory cytokines release. The compound is a scavenger of radical activity, a strong inhibitor of intracellular ROS generation, and an enhancer of the cellular antioxidant system. It has been demonstrated that pretreatment of the cells with cyanidin improved the expression of antioxidant enzymes, namely SOD, CAT, and GPx [219,220,221]. Moreover, Kaewmool et al. showed that, in LPS-stimulated BV2 microglia, cyanidin (2.5–10 mM) inhibits the signaling pathways NF-κB and p38, while MAPK suppresses the production of interleukin-1β (IL-1β) and interleukin-6 (IL-6) and subregulates the gene expressions of iNOS and COX-2 in BV2 cells [222]. In Table 1, the reported data from the in vitro and in vivo studies are described.

Unfortunately, there is still a lack of translational research and clinical evidence for these promising compounds, and we found only one clinical trial, which began in 2022 and will finish in 2024, studying the efficacy and safety of the Flos Gossypii flavonoid tablet in the treatment of Alzheimer’s disease [223]. A total of 240 patients (male and female), aged between 50 to 85 years old, who meet the diagnostic criteria of “likely AD dementia” of the National Institute on Aging—Alzheimer’s Disease Association, are primary school graduates/graduates and above, and have the ability to complete the cognitive ability test and other tests specified in the program will be enrolled. The study proposed a multicenter, randomized, double-blind, placebo-controlled, parallel method to recruit AD patients in order to confirm the efficacy and safety of the Flos Gossypii Flavonoid Tablet. This Phase II clinical trial aims to demonstrate the efficacy and safety of the Flos Gossypii Flavonoid Tablet in the treatment of mild to moderate Alzheimer’s disease (marinus sea deficiency/brain collateral stasis syndrome). The study will monitor changes in AD patients’ general cognitive and daily living activities, different cognitive domain functions, and symptom gravity. The Primary Outcome Measure is the assessment of the Alzheimer’s Disease Scale—Cognitive section (ADAS-cog/11) based on the change from baseline ADAS-cog scores to those at Week 26. It also checks the differences between the low-dose and high-dose groups in the changes in ADAS cog/11 scores (relative to baseline) at weeks 13 and 26, when compared to the placebo group. Seven components will be utilized for the assessment of ADAS-cog cognitive function: word recall, instruction, structural practice, naming, conceptual practice, orientation, and word recognition. The total score ranges from 0 to 70, with lower scores representing milder disease progression. The secondary outcomes include: mini-mental state examination, Alzheimer’s disease co-operative study—activities of daily living, clinician interview-based impression of severity, neuropsychiatric inventory, and dementia syndrome classification scale.

## 4. Conclusions

This review provides evidence that flavonoids have potential for treating AD, and are considered drug candidates for future clinical research. Although precise mechanisms are still unclear, flavonoids regulate several important physiological responses, which may contribute to neuroprotective effects in AD. The advantage of flavonoids over conventional targeting drugs is the possibility of administering these molecules as food supplements. Supplementation with flavonoids could allow for early protection, even at a young age. They can also be used without the need for a preclinical diagnosis, due to their low toxicity. Certainly, further long-term dietary intervention studies indicating the dosage and the times of drug assumption may contribute to fully evaluating the effectiveness of flavonoids as agents for the management of AD. It will be important to incorporate bioavailability and metabolism into experimental planning at all stages of preclinical research, in order to better clarify such mechanisms in vivo.

## Figures and Tables

**Table 1:** Preclinical studies of flavonoids and their neuroprotective role against Alzheimer’s disease.

Flavonoids	Molecular Targets	Model	Dose	References
**Quercetin**	Regulates MAPK signaling	HT22 cells	5 μmol/L	[103]
	Decreases phosphorylation of Tau protein	HT22 cells	5–10 μmol/L	[104]
	Reduces apoptosis and caspase 3 activation	PC-12 cell line	500 μM	[108]
	Restores antioxidant cellular defenses	Gerbilli’s CA1 pyramidal neurons, HepG2 cells, C57BL/6J mice	20 mg/kg, 50 μM 1% quercetin diet	[113,114,115,116]
	Inhibits AChE and BChE	AChE (EC 3.1.1.7 Sigma) BChE (EC 3.1.1.8, Sigma)	1 mg/mL	[118,119]
**Naringerin**	Decreases phosphorylation of Tau protein	PC12 cells	400 μM	[128,129,130]
	Reduces apoptosis and caspase 3 activation	Rat pups	50–100 mM	[128,129,130,131]
	Decreases the inflammatory pathway	Male rats, glial cells	20 mg/kg/day, 0.1–0.3 μmol/L	[132,133,134,135]
	Regulates the MAPK signaling pathway	BV-2 microglial cell line	100 mM	[136]
	Improves the antioxidant system	C57BL/6J mice	25–100 mg/kg	[137,138,139,140]
**Epigallocatechin-3-gallate**	Reduces the accumulation of b amyloid	mice P8 (SAMP8), SweAPP N2 a cells, mouse model, MC65 cells	5–15 mg/kg/day, 20 mM, 1–3 mg/kg, 5–20 μM	[144,145,146,147,148]
	Restores antioxidant cellular defenses	EOC 13.31 microglial cell line, chicken lymphocytes	5–20 μM, 22.5–90 μM	[149,150]
**Myricetin**	Improves learning and memory	Rat models	5 or 10 mg/kg	[160]
	Decreases Ab aggregation	[156,157,158,159,160]		[162,163,164,165,166]
	Regulates a and b secretase activity	rat primary cortical neurons	10 μM	[166]
	Inhibits oxidative stress	Murine models	40–80 µM	[158,169,170]
**Gossypetin**	Inhibits lipid peroxidation	Murine macrophage cell line J774A.1	1–1000 μM	[182]
	Fights against Atherosclerosis	vascular smooth muscle cells	0.1–0.5 μM	[183]
**Genistein**	Reduces the production and deposition of Ab aggregates	Rat model	10 mg/kg	[187,188,189]
	Prevents Tau hyperphosphorylation	Rat model	10 mg/kg	[190]
	Regulates a and b secretase activity	Rat hippocampal neurons	0.375 µg/mL	[194]
	Prevents neuro-inflammation	RAW 264.7 cell model	20 μM	[199]
**Apigenin**	Reduces Tau hyperphosphorylation	Rat model	50 mg/kg	[203]
	Inhibits the production of IL-6 and IL-1b	Human THP-1 monotypic cells	25 µM	[204,205,206]
**Cyanidin**	Regulates NF-κB and p38 MAPK signaling pathways	LPS-stimulated BV2 microglia	2.5–10 mM	[220]

## Data Availability

Not applicable.

## Abbreviations

Alzheimer disease (AD);
Presenilin1 (PS1);
Presenilin 2 (PS2);
Amyloid Precursor Protein (APP);
Apolipoprotein E (ApoE);
Amyloid beta peptide (Aβ);
Intraneuronal Neurofibrillary Tangles (NFTs);
Central Nervous System (CNS);
malondialdehyde (MDA);
4-hydroxynonenal (4-HNE);
Mild Cognitive Impairment (MCI);
Reactive Oxygen Species (ROS);
Carbonyl formation 3-nitrotyrosine (3-NT);
superoxide dismutase (SOD);
red blood cells (RBC);
Reactive Nitrogen Species (RNS);
AMP Activated Protein Kinase (AMPK);
Glycogen Synthase Kinase 3 beta (GSK3β);
cyclin-dependent kinase 5 (cdk5);
Protein Phosphatase 2A (PP2A);
nitric oxide synthase (NOS);
Peroxisome proliferator-activated receptor-γ coactivator (PGC-1α);
glutathione (GSH);
catalase (CAT);
glutathione peroxidase (GPx);
Acetylcholinesterase (AchE);
Butyrylcholinesterase (BchE);
Acetylcholine (ACh);
nuclear factor-kB (NF-kB);
tumor necrosis factor-α (TNF-α);
interleukin (IL);
sirtuin 1 (SIRT1);
inducible nitric oxide synthase (iNOS);
cyclooxygenase 2 (COX-2);
glutathione reductase (GR);
hydrogen peroxide (H_2_O_2_);
Epigallocatechin-3-gallate (EGCG),
blood–brain barrier (BBB).
